# Clinical Outcomes after International Referral of Uveal Melanoma Patients for Proton Therapy

**DOI:** 10.3390/cancers13246241

**Published:** 2021-12-13

**Authors:** Marina Marinkovic, Lennart J. Pors, Vincent van den Berg, Femke P. Peters, Ann Schalenbourg, Leonidas Zografos, Alessia Pica, Jan Hrbacek, Sjoerd G. Van Duinen, T. H. Khanh Vu, Jaco C. Bleeker, Coen R. N. Rasch, Martine J. Jager, Gregorius P. M. Luyten, Nanda Horeweg

**Affiliations:** 1 Department of Ophthalmology, Leiden University Medical Center, 2333 ZA Leiden, The Netherlands; M.Marinkovic@lumc.nl (M.M.); vvdberg8980@gmail.com (V.v.d.B.); T.H.K.Vu@lumc.nl (T.H.K.V.); j.c.bleeker@lumc.nl (J.C.B.); m.j.jager@lumc.nl (M.J.J.); g.p.m.luyten@lumc.nl (G.P.M.L.); 2 Department of Radiation Oncology, Leiden University Medical Center, 2333 ZA Leiden, The Netherlands; l.j.pors@lumc.nl (L.J.P.); f.peters@nki.nl (F.P.P.); C.R.N.Rasch@lumc.nl (C.R.N.R.); 3 Department of Ophthalmology, University of Lausanne, Jules-Gonin Eye Hospital, FAA, 1004 Lausanne, Switzerland; ann.schalenbourg@fa2.ch (A.S.); zogcab@gmail.com (L.Z.); 4 Proton therapy Center, Paul Scherrer Institute, ETH Domain, 5232 Villigen, Switzerland; Alessia.pica@psi.ch (A.P.); jan.hrbacek@psi.ch (J.H.); 5 Department of Pathology, Leiden University Medical Center, 2333 ZA Leiden, The Netherlands; S.G.van_Duinen@lumc.nl

**Keywords:** uveal melanoma, proton therapy, organ preservation, neoplasm recurrence, local, survival, visual acuity

## Abstract

**Simple Summary:**

This study aims to assess cancer control and preservation of the eye and visual acuity after proton therapy abroad for eye melanoma. For this, medical files were reviewed of Dutch uveal melanoma patients who were treated in Switzerland with proton therapy from 1987 to 2019. The tumours of these patients were too large and/or localised too close to the optic nerve to be treated with local plaque irradiation. There were 103 patients, of whom one had a uveal melanoma in both eyes. The tumours were relatively large and often localised around the central part of the retina. At five years after treatment, proton therapy had controlled the uveal melanomas of 94% of the patients and 81% had preserved their eye. Spread of the cancer beyond the eye was observed in 30% of the patients. Most patients (79%) became blind or had severe visual impairment after proton therapy; a small group of patients had mild or no visual impairment (17%). The size of the tumour, its localisation and the dose of proton therapy were important for the risk of decline in visual acuity. This study shows that proton therapy abroad for uveal melanoma is feasible and yields good results.

**Abstract:**

Objective: To assess oncological and ophthalmological outcomes after international referral of uveal melanoma patients for proton therapy. Materials and Methods: This is a retrospective study among Dutch uveal melanoma patients who were treated in Switzerland with 60.0 CGE proton therapy (in 4 fractions) from 1987 to 2019. All patients were ineligible for brachytherapy due to tumour size and/or proximity to the optic nerve. Time-to-event analyses were performed using Kaplan–Meier’s methodology and Cox proportional hazards models. Results: There were 103 patients (104 eyes) with a median largest tumour diameter of 19 mm (range 6–26 mm). Tumours were localised centrally (11%), mid-peripherally (65%) or peripherally (34%). Median follow-up was 7 years. Five-year local control, distant metastasis-free survival and eye preservation rates were 94%, 70% and 81% respectively. At five years, severe, moderate and mild visual impairment was observed in respectively 79%, 4% and 6% of the patients. Larger tumour volumes and more central tumour localisation were associated with severe visual impairment. After correction for these factors, dose to the macula, optic disc and retina, but not optic nerve was significantly associated with severe visual impairment. Conclusion: International referral for proton therapy yielded good tumour control and eye preservation rates, but risk of distant metastasis and severe visual impairment were substantial, possibly due to the selection of advanced tumour stages and/or central localisation. Dose to the macula may be more relevant than dose to the optic nerve for preservation of visual acuity, which is relevant for the treatment planning of proton therapy.

## 1. Introduction

Uveal melanoma is a rare malignancy with an incidence of 1–9 cases per million per year [1,2]. Treatment modalities include enucleation, brachytherapy or external beam radiation therapy, either stereotactic with photons or with charged particles, such as protons [3,4]. Radiotherapeutic techniques have similar efficacy as enucleation for reducing risk of metastasis and death [5,6,7,8,9]. Brachytherapy, especially with ruthenium episcleral plaques, is associated with the least visual complications and is relatively affordable compared to charged particle therapy [10,11]. However, if brachytherapy is technically not possible due to tumour size or involvement of the optic nerve, proton therapy is an attractive alternative to enucleation. With proton therapy, eye preservation is reported to be achieved in 75–95% of patients [12,13,14,15,16,17,18,19], and preservation of visual acuity ≥ 0.1 (Snellen ≥ 6/60, logMAR ≥ 1.0) in 46–69% at 5 years [13,14,19,20,21]. Four research groups demonstrated that proton therapy also yields good outcomes in patients with juxtapapillary melanomas [21,22,23].

If proton therapy is not available, patients must choose between travelling long distances or undergoing photon radiotherapy or enucleation locally. Travelling may be worthwhile, as proton therapy offers superior sparing of the healthy eye tissues around the tumour [24]. However, it is unclear whether patients who undergo proton therapy at distant centres have clinical outcomes as favourable as those published. Patients might face relatively long waiting times due to increased administrative and logistic burden. In addition, they may have relatively large tumours, because those with tumour sizes that are borderline for brachytherapy may not be offered proton therapy.

In this study, we evaluated the clinical outcomes of Dutch uveal melanoma patients who were referred for proton therapy to Switzerland. Our results provide realistic estimates of tumour control, eye preservation and visual acuity after international referral. These estimates may inform physician and patients to decide whether referral for proton therapy to distant centres is worthwhile.

## 2. Materials and Methods

### 2.1. Patients

This is a retrospective study of uveal melanoma patients from the Leiden University Medical Centre (LUMC) in the Netherlands who were ineligible for brachytherapy. All patients were referred to the Jules–Gonin Eye Hospital (JGEH) in Lausanne and treated with proton therapy at the Paul Scherrer Institute (PSI) in Villigen, Switzerland from 1987 to 2019.

At LUMC before referral to Switzerland, all patients were diagnosed using slit lamp examination, fundus photography and, if necessary, fluorescein angiography (FAG). The largest basal diameter and height of the tumour were determined by ultrasonography. Screening for distant metastases was performed by liver ultrasonography, chest radiography and liver function tests. All patients were discussed at the institutional multidisciplinary eye oncology meeting (ophthalmology, pathology, radiation oncology, and medical physics). According to the LUMC uveal melanoma treatment protocol, proton therapy was offered to patients if their tumours were > 7 mm in height and/or > 16 mm in diameter and/or had > ⅓ involvement of the circumference of the optic disc [11]. Patients not willing or able to undergo proton therapy abroad underwent enucleation and were not included in this study. Patients with metastatic disease at time of diagnosis were also excluded from this study.

### 2.2. Treatment Procedure

A detailed description of the treatment procedures is published [25]. Briefly, patients were examined by an expert ophthalmologist at JGEH (LZ, AS) and underwent surgical placement of 3–7 tantalum clips on the outer surface of the sclera to mark the tumour base. Thereafter, patients went to the PSI for preparation of proton therapy, consisting of crafting a custom-moulded head holder with a bite-block, and a simulation procedure wherein orthogonal X-rays were taken in treatment position with fixed gaze to determine tumour localisation using the tantalum clips [25]. Proton therapy was planned, using modified EYEPLAN planning software [26]. EYEPLAN builds a two-sphere model of the eye (including organs at risk) using clip coordinates and ultrasound eye length. Into this model, the shape of the tumour base (defined by clip positions and fundus photographs) and tumour profile (based on B-scan ultrasound) were drawn. The treatment position of the eye was chosen to achieve complete tumour irradiation with a 2.5 mm margin, while minimizing dose to the optic disc and nerve, macula, ciliary body, and lens in descending priority. All patients received 60 Cobalt Gray Equivalent (CGE) in four fractions on four consecutive days.

### 2.3. Follow-up

All patients were followed up after proton therapy at LUMC at 1, 4, 7 and 12 months and twice a year thereafter for ≥5 years. At each visit, tumour response (by fundoscopy, ultrasound and if needed FAG), visual acuity (best corrected by Snellen chart) and side-effects were assessed. From 2012 onwards, bevacizumab injections for the prevention of neovascular glaucoma was the standard of care [27]. Biannual screening for liver metastasis by ultrasonography was performed, followed by MRI in case of abnormalities. Patients with metastasis were referred to a medical oncologist; continuation of ophthalmological follow-up depended on the patient’s physical condition and presence of eye symptoms.

### 2.4. Data

Data were retrieved from electronic or scanned paper patient files from 1987 until July 2020. Data on tumour recurrence, metastasis, and cause of death were collected from the patient’s files and verified via the National Dutch Cancer Registry.

**Figure 1:**
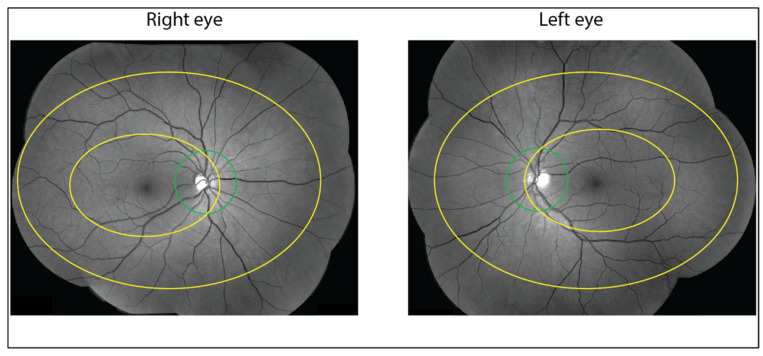
**Relation between visual acuity after proton therapy and dose parameters by tumour localisation.** Tumour localisation is done by classifying each tumour into central, mid-peripheral or peripheral localisation by considering the most centrally localised part of the tumour. Central tumours are localised within the temporal retinal arcade indicated by the small yellow oval. Mid-peripheral tumours are localised outside of this vascular arcade and are readily visible by indirect fundoscopy, indicated by the space between the small and big yellow oval. Peripheral tumours are localised further beyond and may be visualised by fundoscopy at angle or with the help of a mirror lens, indicated by the space outside the big yellow oval. Juxtapapillary tumours are defined as being localised within the green circle around the optic disc that extends 1 optic disc diameter beyond the edge.

For this study, tumour localisation was reassessed by an expert ophthalmologist (MM) using the description in the patient file, fundus photographs and ultrasound reports at the time of diagnosis. Tumour localisation was classified as central, mid-peripheral or peripheral localisation by considering the most centrally localised part of the tumour. Tumours were classified as central if localised within the temporal retinal arcade (Figure 1). Tumours were classified as mid-peripheral if localised outside of the vascular arcade, but readily visible by indirect fundoscopy. Tumours were classified as peripheral if localised further beyond and visualisation was not possible or only possible by fundoscopy at angle or with the help of a mirror lens. Tumours were classified as juxtapapillary if localised within 1 optic disc diameter beyond the edge of the optic disc.

For analysis, we used the tumour height determined by B-scan ultrasonography during the pre-operative examination at JGEH. The tumour volume was estimated by the EYEPLAN software based on the tumour height, diameter, shape (defined by clip positions and fundus photographs) and tumour profile (based on B-scan ultrasound). Dosimetric parameters (D90, D50, and D20) for eye structures at risk were estimated by the EYEPLAN software. Prior to the analyses, it was defined to use the D50 for investigation of the relation between dose and loss of visual acuity. We used the visual acuity measurement performed at JGEH just before proton therapy as the baseline assessment of visual acuity for analyses.

### 2.5. Definitions

This study evaluates the time to (uveal melanoma-specific) death, distant metastasis, local failure, secondary enucleation and loss of visual acuity. All times were calculated from the first day of proton therapy to the day of the event of interest or last day of follow-up in patients without events. Time to start of treatment was calculated from the first visit at LUMC for (suspected) uveal melanoma to the first fraction of proton therapy.

Local failures were diagnosed by the treating LUMC expert ophthalmologist (MM, VK, JB, GL) if (i) the tumour had regressed or disappeared after treatment and showed significant (re)growth; (ii) the tumour showed significant continuous growth after proton therapy; or (iii) the tumour remained stable in size after proton therapy and had signs suspicious of recurrence (new pinpoint leakages on FAG or decreased reflectivity on ultrasound) at follow-up examinations after 6 months. An expert pathologist (SvD) reviewed all specimens of the eyes enucleated for recurrences and toxicity to confirm the local failures and to assess histopathological features, such as mitotic figures, of the vital tumour residue.

Visual acuity was categorised according to the International Classification of Diseases version 11 into severe visual impairment (<0.1, Snellen < 6/60, LogMAR > 1.0), moderate visual impairment (0.1 to <0.3, Snellen 6/60 to <6/20, LogMAR 1 to >0.5) and mild visual impairment (<0.5, Snellen 6/20 to <6/12, LogMAR 0.5 to >0.3) [28].

### 2.6. Statistical Methods

Median follow-up was estimated using the reverse Kaplan–Meier methodology [29]. Time to event analyses were performed using Kaplan–Meier’s methodology and Cox’ proportional hazards models. Missing measurements of visual acuity between available measurements were imputed within cases using linear interpolation. Patients who underwent secondary enucleation were defined to have severe visual impairment (<0.1, Snellen < 6/60, LogMAR > 1.0) until the end of follow-up or death. Multivariable regression analyses were performed by forced entry of significant risk factors identified by univariable analysis, respecting the rule of thumb that around 10 events are required per predictor. Statistical analyses were performed using SPSS (Version 26). All *p*-values are two-sided. Statistical significance was accepted at *p*-values < 0.05.

Hierarchical clustering of cases according to visual acuity was done according to Ward’s minimum variance method with Euclidean distances [30] stratified by tumour localisation. Doses (D50) to the macula, retina, optic disc were added to the cluster heat map as non-clustering annotation columns. Analyses were performed in R version 3.6.1. (http://www.r-project.org/; accessed on 10 December 2021) using the ComplexHeatmap package.

## 3. Results

A total of 103 patients with 104 affected eyes were included for analysis. Patient, tumour and treatment characteristics are presented in Table 1. All 104 tumours had one or more eligibility criteria for proton therapy (juxtapapillary localisation, tumour height > 7 mm, and/or diameter > 16 mm). Median time to start of treatment was 42 days (range 14–175 days), 80% of patients were treated within 60 days. Median follow-up was 7.0 years (range: 0.0–24.1 years).

### 3.1. Oncological Outcomes and Eye Preservation

During follow-up, 42 of 103 patients passed away: 31 due to uveal melanoma (73.8%), 8 due to intercurrent disease (19.0%) and in 3 patients (7.1%), the cause of death was unknown. Metastasis-free survival and overall survival were, respectively, 70.2% and 67.7% at five years after proton therapy (Table 2). Symptoms or routine follow-up examination led to diagnosis of a local failure in 8 of 104 eyes (7.7%). Local control rate was 94.3% at five years after proton therapy (Table 2). Seven of these eight patients with local failure underwent secondary enucleation; one did not receive local treatment because he was simultaneously diagnosed with liver metastases. Pathology review confirmed the diagnosis of local failure in the seven enucleated eyes. Kaplan–Meier curves for local recurrence-free and distant metastasis-free survival are provided in Figure S1.

In addition to the seven enucleations for local failure, 15 enucleations (14.4%) were performed for treatment toxicity: neovascular glaucoma (*n* = 3), retinal detachment (*n* = 2), rubeosis (*n* = 1), blind and painful eyes (*n* = 2), neovascular glaucoma and retinal detachment (*n* = 3), retinal detachment and rubeosis (*n* = 1), rubeosis and pain (*n* = 1) and vitreous haemorrhage and blindness (*n* = 2). Pathology review showed in 12 of these 15 (80%) resections specimens a tumour residue with viable cells and/or mitotic figures. Most of these 12 patients were enucleated shortly after proton therapy: <1 year (*n* = 3), 1–2 years (*n* = 7) and >2 years (*n* = 2). The five-year eye preservation rate was 81.3%; the Kaplan–Meier curve is provided in Figure S2.

### 3.2. Preservation of Visual Acuity

Visual acuity before and after proton therapy is presented in Table 3. Most of the decline in visual acuity was observed in the first two years after treatment. At five years after proton therapy 78.9% of the patients had severe visual impairment (visual acuity < 0.1, Snellen < 6/60, logMAR < 1.0). The Kaplan–Meier curve of severe visual impairment-free survival is provided in the supplemental data (Figure S2).

**Figure 2:**
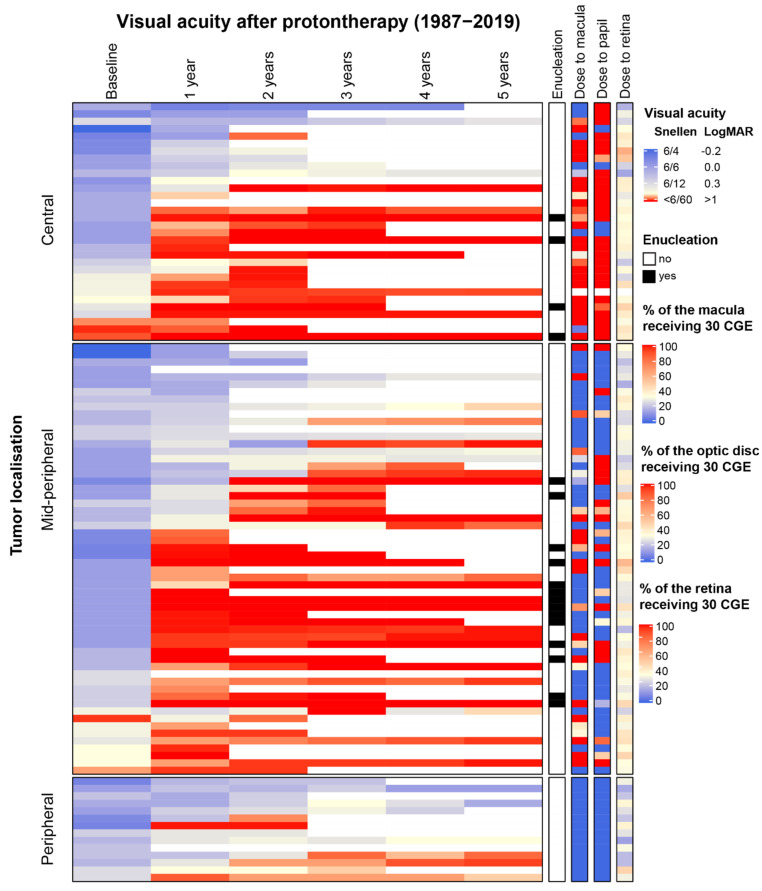
**Relation between visual acuity after proton therapy and dose parameters by tumour localisation.** Each horizontal row in the heat map represents an eye. The total number included eyes in this analysis is 103. Data is clustered on visual acuity during follow-up by tumour localisation. The four non-clustering annotations bars at the right side of the heat map display the values for each eye for enucleations in the first 5 years and three dose parameters. This graph gives on overview of the pattern of decline in visual acuity across follow-up in the cohort and shows the relation between visual impairment and relevant clinical and radiotherapeutic factors at the individual level.

Clustering of visual acuity by tumour localisation is shown in Figure 2. Each eye (*n* = 104) is represented in the heat map by a row that indicates visual acuity from baseline until five years after proton therapy. The annotation bars on the right side of the graph show the relation of visual acuity with dose to the organs at risk, which seems strongest for dose to the macula (Figure 2). This graph also shows that improvement of visual acuity is unlikely if it is low or absent before proton therapy. Remarkably, a few patients who had received 60 CGE to the optic disc preserved good visual acuity. All these patients had very small juxtapapillary tumours and relatively low doses to the macula.

The risk factors for severe visual impairment identified by univariable regression analyses are presented in Table 4. These analyses confirmed the importance of tumour volume and localisation and showed that retinal detachment before proton therapy is associated with a two-fold risk of severe visual impairment. Further, analyses showed that a dose of 30 CGE (D50) to the macula, retina, optic disc, but not optic nerve, were significantly associated with severe visual impairment. Possibilities to perform multivariable analysis were limited by the numbers of eyes included in this study (only 34 had a visual acuity of >0.1). Therefore, four separate multivariable Cox proportional hazards models were built, which always included tumour volume and localisation, and a dose to the macula, optic disc, optic nerve or retina. These analyses showed that dose (D50, 30 CGE) to the macula (HR 1.007 per %, 95% CI 1.002–1.013, *p* = 0.010), optic disc (HR 1.008 per %, 95% CI 1.002–1.013, *p* = 0.010) and optic nerve (HR 1.145 per mm, 95% CI 01.022–1.284, *p* = 0.020), but not the retina (HR 1.026 per %, 95% CI 0.991–1.063, *p* = 0.14) was significantly and independently associated with severe visual impairment. Collinearity of the dose to parameters precluded further analysis to determine which of these dose parameters is independently associated with severe visual impairment.

## 4. Discussion

In this study, we evaluated the clinical outcomes of Dutch uveal melanoma patients who were ineligible for brachytherapy and referred to Switzerland for proton therapy between 1987 and 2019. At 5 years after treatment, 94.3% of the patients were free of local failure, 81.3% had preserved their eye and 78.9% had severe visual impairment.

### 4.1. Oncological Outcomes

Local control after proton therapy among our patients (94% at 5-years) was comparable to that in the literature (91–97%) despite their relatively large tumour sizes (median 19 mm) [12,13,14,15,16,25,31]. Our pathology review of all enucleated eyes (both for local failure and toxicity) showed that tumour residues with vital cells and mitotic figures were present regardless of the presence of a true local failure. It is known that a dose of 60 CGE by proton therapy does not cause necrosis of the tumour, but rather sterilises tumour cells via senescence and mitotic catastrophe [32]. These processes lead to cell death after several replications [32]. Therefore, tumour regression is slow, and some parts may look vital years after proton therapy [33]. Hence, the presence of vital cells and mitotic figures should not be interpreted as treatment failure. Nonetheless, ophthalmologists should be cautious if patients suffer from progressive toxicity because this may be caused by a growing tumour in some cases.

At five years after treatment, 30% of our patients were diagnosed with distant metastases, compared to 19–25% in published series [12,14,15,34,35]. The relatively large tumour size and high prevalence of extrascleral tumour extension (6.7% vs. 4.5% [3] and 3.3% [26]) possibly contributes to this difference. Overall survival of our patients (68% at five years) was in the range of reported estimates (52–88%) [6,12,14,15,20,21,25,35].

### 4.2. Eye Preservation

A total of 81% of our patients and 75–95% of the patients in other studies had preserved their eye at 5 years after treatment [12,13,14,15,16,17,19,25]. Secondary enucleation was performed for neovascular glaucoma (sometimes in combination with other toxicities) in 40% of our patients, which seems in line with the 30–75% reported by others [12,13,14,17,20,25,35,36]. Another common reason for secondary enucleation in our cohort was retinal detachment (40%, sometimes with other toxicities), which was also reported in other studies, though less frequently (12–14%) [13,17]. This may be explained by the relatively large tumour size in our cohort. Also, about half of our cohort was treated before the introduction of routine administration of intravitreal bevacizumab, which reduces risk of neovascular glaucoma [27,37].

Future studies may identify risk factors for secondary enucleation, enabling more selective allocation of proton therapy or enucleation as primary treatment. However, this should be accompanied by research on preferences of patients for primary enucleation versus proton therapy with a high risk of toxicity and secondary enucleation [38].

### 4.3. Preservation of Visual Acuity

Our analysis showed that the decline in visual acuity after proton therapy is substantial in the first two years and becomes steadier thereafter. This observation by us and others [39] might correspond to ‘early’ toxicities with a high risk of visual impairment, such as macular oedema and retinal detachment, and toxicities that progress slowly, such as radiation maculopathy, papillopathy, retinopathy and neovascular glaucoma. Our longitudinal analysis showed some fluctuations in visual acuity over time. This is possibly caused by transient or treatable toxicities such as cataract, which adds a level of complexity to the analysis of visual acuity after proton therapy.

Severe visual impairment (<0.1, Snellen < 6/60, LogMAR > 1.0) was observed in 39% of our patients after 1 year, increasing to 65% at 2 years and to 79% at five years. The prevalence of severe visual impairment at 5 years was 46–69% in the series published by proton therapy centres [13,14,19,20,21]. Factors such as our referral criteria for proton therapy (large tumours and/or juxtapapillary localisation), lack of prophylactic bevacizumab and a protocol to prevent radiation maculopathy probably contributed to the worse visual outcomes our cohort. Considering these practice changes, visual outcomes are likely to be better for patients who are currently treated in Switzerland.

When comparing visual outcomes between cohorts, differences in risk factors should be considered. This is not straightforward, as the reporting of risk factors is not uniform and often unclear. We classified tumour localisation according to a simple system (Figure 1 and Figure 2) in this study. We also use it in our clinical practice for all patients that undergo brachytherapy and proton therapy, which has been performed at the Holland Particle Therapy Centre in Delft since 2020.

Risk factors for loss of visual acuity identified by us and others include tumour size [13,20,21,40,41,42], pre-treatment visual acuity [13,15,21,31,40,42] and retinal detachment [15,40], a more central tumour localisation [15,20,23,39,40,43] and dose to the macula [15,21,23,40,42], retina [42] and optic disc [40,42]. Based on our and the aforementioned studies, dose to the macula/fovea seems to have a stronger association with loss of visual acuity than dose to the optic disc/nerve. This hypothesis is supported by a number of studies that showed that high doses to the optic disc and nerve do not necessarily result in loss of vision or radiation optic neuropathy [23,42,43]. However, this finding could also be explained by discrepancies between planned and delivered dose due to the current planning technique that relies on a standard model of the eye and derivatives for tumour localisation. Current proton therapy planning strategies often prioritise sparing of the optic disc and nerve over sparing of the macula. It is complicated to isolate the impact of dose parameters of eye structures that are in proximity, and it is therefore hard to determine whether the current planning strategy is suboptimal or not. Encouraging developments in the field of magnetic resonance imaging of the eye may enable more precise proton therapy planning in the near future [44,45,46].

### 4.4. Limitations

Selection bias and registration bias are inherent to the retrospective design of this study, which may have led to an underestimation of the incidence of the events reported in this study. We expect this to be minimal because we cross-checked oncological outcomes and survival in the National Dutch Cancer Registry. The interpretation of the comparisons between our cohort other cohorts should be done with caution. The patient selection is different in each study, even if Dutch and Swiss patients treated in Switzerland would be compared [18,23,25,47].

### 4.5. Implications

Proton therapy is increasingly available for the treatment of uveal melanomas and is a valuable alternative for enucleation. Proton therapy offers good local tumour control, eye preservation and for some patients, even visual acuity preservation, which is important for functioning and quality of life [48]. Future studies should aim to further improve visual outcomes and reduce side effects. The integration of high-resolution imaging in proton therapy treatment planning will be a key step in this journey [45]. This and other studies showed that distant metastasis are common and impair overall survival. Unfortunately, there are currently no systemic therapies that are recommended in uveal melanoma [49]. Several clinical trials in the adjuvant and metastatic setting are ongoing and will hopefully change this in the near future [50].

## 5. Conclusions

International referral of uveal melanoma patients for proton therapy is feasible and yielded good local control and eye preservation. However, risk of distant metastasis and severe visual impairment were substantial, which may be explained by our referral criteria based on large tumour size and central tumour localisation. These results may help physicians and patients to decide whether proton therapy at distant centres is worthwhile. Our analyses indicate that sparing of the macula may be more important for prevention of severe visual impairment than sparing of the optic disc and nerve, which is relevant for the further development of proton therapy treatment planning.

## Figures and Tables

**Table 1:** Patient, tumour and treatment characteristics.

Patient and Tumour Characteristics	*n* (%)
No. of patients	103 (100.0)
Age in years—mean (range)	59 (24–85)
Gender—male	55 (53.4)
Diabetes mellitus	8 (7.8)
No. of eyes	104 (100.0)
Right eye affected	59 (56.7)
Visual acuity—median (range)	0.90 (0.01–1.50)
Tumour diameter in mm—median (range)	18.7 (6.4–25.7)
Tumour height in mm—median (range)	8.4 (1.5–17.7)
Tumour volume in mm^3^—median (range)	1163 (28–3369)
Extrascleral tumour extension	7 (6.7)
Tumour localisation	
central	11 (10.6)
mid-peripheral	68 (65.4)
peripheral	25 (24.0)
Juxtapapillary localisation	35 (33.7)
Tumour stage	
T1	3 (2.9)
T2	9 (8.7)
T3	27 (26.0)
T4	65 (62.5)
**Planned doses (total dose 60 CGE)**	**median (range)**
D50 tumour (in %)	100 (100–100)
D50 retina (in %)	35 (14–63)
D50 macula (in %)	5 (0–100)
D50 optic disc (in %)	0 (0–100)
D50 optic nerve (in mm)	0.0 (0.0–9.8)
D50 ciliary body (in %)	31 (2–67)
D50 lens (in %)	21 (0–89)

Definition of abbreviations: CGE = cobalt grey equivalent; D50 = volume that has received 50% of the total prescribed dose (60 CGE for all patients in this study). Applied margins: anterior median 5.9 mm (range −11.3–14.6), distal median 2.5 mm (range 2.2–4.0), proximal median 2.5 mm (range 0–5.0).

**Table 2:** Survival, local tumour control and eye preservation after proton therapy for uveal melanoma.

Events/Cases		Actuarial Estimates (SE)				Time to Event
		1 Year	3 Years	5 Years	7 Years	Median (SE)
Overall survival	42/103	97.0% (1.7)	83.6% (4.1)	67.7% (5.4)	61.0% (5.8)	10.0 (2.2)
Disease-specific survival	31/100	96.9% (1.7)	85.5% (3.9)	71.5% (5.4)	64.1% (6.0)	17.7 (6.0)
Distant metastasis-free survival	32/103	93.8% (2.4)	81.8% (4.2)	70.2% (5.4)	62.0% (6.1)	17.7 (5.7)
Local control	8/104	96.8% (1.8)	94.3% (2.5)	94.3% (2.5)	91.6% (3.6)	not reached
Eye preservation	22/104	92.9% (2.6)	81.3% (4.3)	81.3% (4.3)	77.4% (4.8)	not reached

Definition of abbreviations: SE = standard error.

**Table 3:** Visual acuity following proton therapy for uveal melanoma.

Events/Cases		Actuarial Estimates (SE)				Time to Event
		1 Year	2 Years	3 Years	5 Years	Median (SE)
Visual acuity < 0.5	83/103	64.5% (5.0)	78.9% (4.4)	86.5% (3.7)	89.2% (3.5)	0.42 (1.00)
Visual acuity < 0.3	76/103	48.5% (5.3)	69.7% (5.0)	76.5% (4.7)	82.6% (4.4)	1.00 (0.26)
Visual acuity < 0.1	69/103	39.2% (5.2)	64.9% (5.3)	75.1% (5.0)	78.9% (4.9)	1.42 (0.25)
**Mild or worse visual impairment by tumour localisation ^1^**						
central	28/32	61.0% (8.8)	83.8% (7.1)	91.9% (5.4)	91.9% (5.4)	0.42 (0.17)
mid-peripheral	46/57	73.1% (6.2)	77.6% (5.9)	84.6% (5.3)	89.7% (4.6)	0.33 (0.10)
peripheral	9/14	44.3% (15.0)	72.1% (11.8)	81.4% (11.8)	81.4% (11.8)	1.08 (0.54)
**Moderate or worse visual impairment by tumour localisation ^2^**						
central	26/32	46.0% (9.1)	80.4% (7.7)	84.3% (7.1)	84.3% (7.1)	1.00 (0.38)
mid-peripheral	44/57	57.9% (7.0)	71.0% (6.6)	80.2% (5.9)	87.6% (5.0)	0.67 (0.25)
peripheral	6/14	21.2% (13.4)	32.5% (15.5)	32.5% (15.5)	46.0% (17.3)	7.83 (3.49)
**Severe visual impairment by tumour localisation ^3^**						
central	24/32	33.5% (8.7)	77.1% (8.5)	86.3% (7.1)	86.3% (7.1)	1.25 (0.35)
mid-peripheral	41/57	48.7% (7.1)	66.3% (6.9)	77.8% (6.4)	80.2% (6.2)	1.25 (0.51)
peripheral	4/14	10.0% (9.5)	21.2% (13.4)	21.2% (13.4)	47.5% (23.2)	NR

Definition of abbreviation: NR = not reached, SE = standard error. ^1^ Visual acuity < 0.5, Snellen 6/12, logMAR 0.3. ^2^ Visual acuity < 0.3, Snellen < 6/20, logMAR 0.5. ^3^ Visual acuity < 0.1, Snellen < 6/60, logMAR 1.

**Table 4:** Risk factors for decline in visual acuity < 0.1 after proton therapy.

Predictors	Hazard Ratio	95% CI	*p*-Value
Age (per year)	1.008	0.990–1.028	0.38
Gender	1.446	0.884–2.364	0.14
Diabetes mellitus	1.525	0.692–3.362	0.30
Pre-treatment visual acuity	0.225	0.268–1.364	0.23
Pre-treatment retinal detachment	2.338	1.242–4.400	0.009
Tumour volume (per mm^3^)	1.000	1.000–1.001	0.019
Tumour diameter (per mm)	1.066	0.995–1.143	0.070
Tumour prominence (per mm)	1.049	0.975–1.130	0.20
T stage			
T1-2	reference		
T3	1.585	0.623–4.030	0.33
T4	1.990	0.840–4.717	0.12
Tumour localisation			
Peripheral	reference		
Mid-peripheral	3.449	1.185–10.044	0.023
Central	3.102	1.103–8.722	0.032
Juxtapapillary localisation	1.357	0.827–2.228	0.23
D50 retina (per %)	1.042	1.016–1.068	0.001
D50 macula (per %)	1.009	1.004–1.015	0.001
D50 optic disc (per %)	1.005	1.000–1.011	0.034
D50 optic nerve (per mm)	0.098	0.984–1.205	0.098
D50 lens (per %)	1.001	0.992–1.011	0.77
Year of treatment	1.025	0.986–1.065	0.21

Definition of abbreviations: CI = confidence interval; D50 = volume that has received 50% of the total prescribed dose (60 CGE for all patients in this study). Risk factors identified by univariable regression analysis using Cox proportional hazards models.

## Data Availability

Data of this study are available via the corresponding author upon reasonable request.

## Supplementary Materials

The following are available online at https://www.mdpi.com/article/10.3390/cancers13246241/s1, Figure S1: Oncological outcomes in all patients, Figure S2: Ophthalmological outcomes in all patients.
