# Supplementary material for: Clinical Outcomes after International Referral of Uveal Melanoma Patients for Proton Therapy
Source: Cancers (Basel). 2021 Dec 13;13(24):6241. doi: 10.3390/cancers13246241 (PMC8699723; doi:10.3390/cancers13246241)
Supplement: Supplementary file 1 [file cancers-13-06241-s001.zip › cancers-1494914-supplementary.pdf]

Supplementary Figure S1. Oncological outcomes in all patients

All patients
Local recurrence-free survival

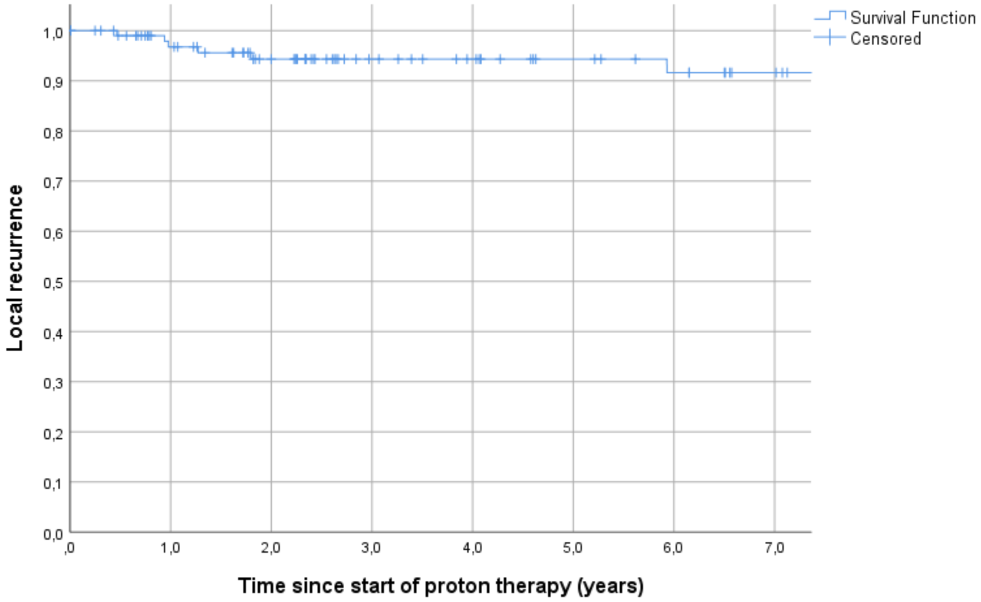

Numbers at risk

**0.0**	**1.0**	**2.0**	**3.0**	**4.0**	**5.0**	**6.0**	**7.0**	years
104	87	70	51	45	38	34	28	

All patients
Distant metastasis-free survival

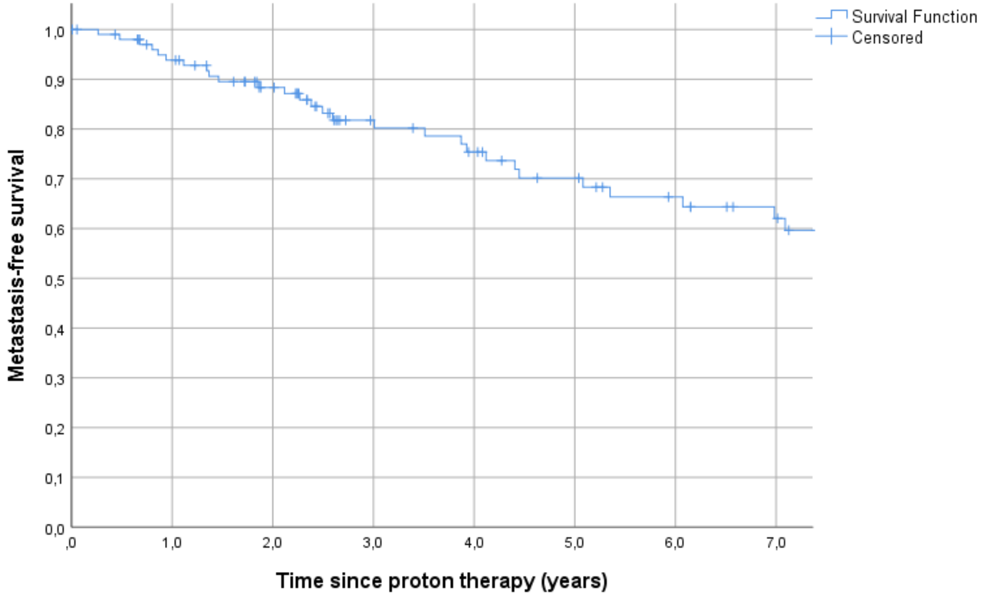

Numbers at risk

**0.0**	**1.0**	**2.0**	**3.0**	**4.0**	**5.0**	**6.0**	**7.0**	years
103	90	74	52	46	39	33	27	

Supplementary Figure S2. Ophthalmological outcomes in all patients

All patients
Enucleation-free survival

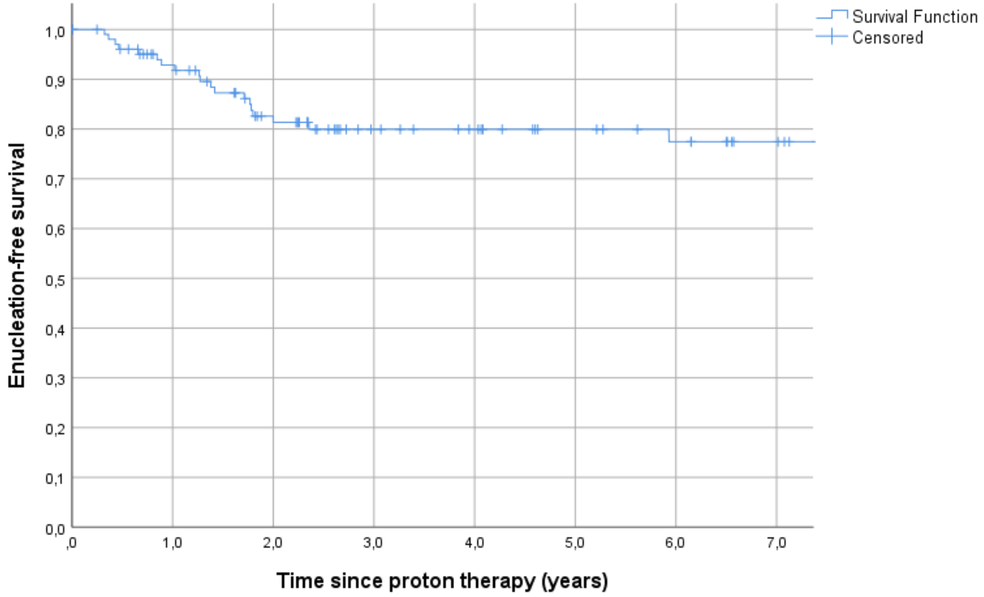

Numbers at risk

**0.0**	**1.0**	**2.0**	**3.0**	**4.0**	**5.0**	**6.0**	**7.0**	years
104	86	66	47	42	35	31	25	

All patients
Severe visual impairment-free survival

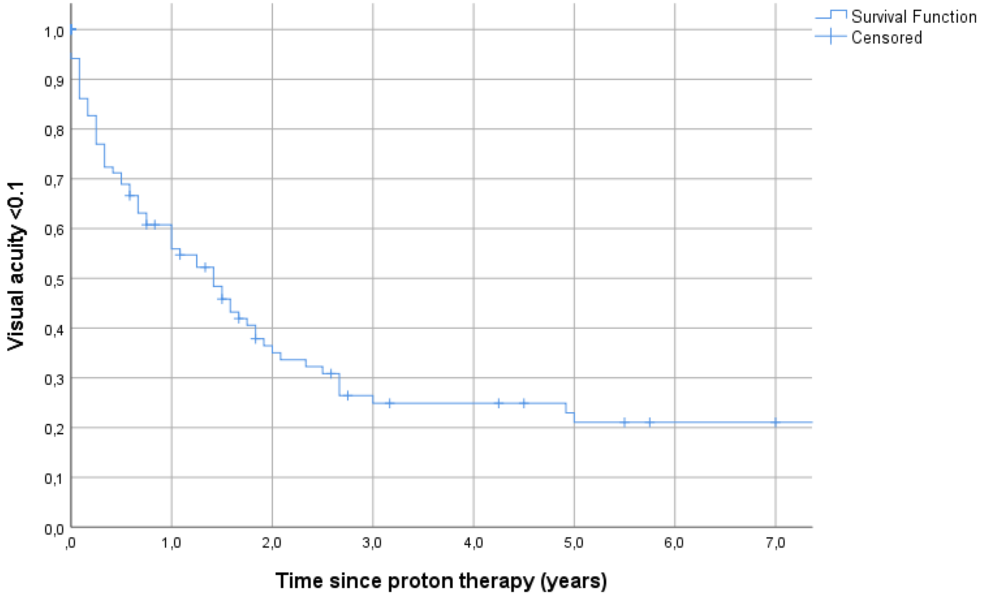

Numbers at risk

**0.0**	**1.0**	**2.0**	**3.0**	**4.0**	**5.0**	**6.0**	**7.0**	years
104	51	27	18	16	13	10	10	
